# The Role of Medication, Mental Illness, and Social Isolation on the Development of the Fetus in the Context of the COVID-19 Pandemic

**DOI:** 10.7759/cureus.48791

**Published:** 2023-11-14

**Authors:** Zayna Z Abdulla, Aleena A Ferozuddin, Angelica Oviedo

**Affiliations:** 1 Physiology & Pathology, Burrell College of Osteopathic Medicine, Las Cruces, USA

**Keywords:** antidepressants, anxiolytics, neonatal withdrawal syndrome, covid-19, maternal medications, benzodiazepines, ssri, maternal mental illness, social isolation

## Abstract

As we enter the COVID-19 post-pandemic period, uncertainty surrounds the impact of the varied effects of medications, mental illness, and social isolation on children born during the pandemic. Medications like selective serotonin reuptake inhibitors (SSRIs) and benzodiazepines during pregnancy, coupled with pandemic-induced social isolation, may contribute to anxiety, depression, and behavioral issues in the offspring. Supporting evidence shows SSRIs' influence on brain development, while third-trimester benzodiazepine use may lead to neonatal withdrawal syndrome. Social isolation during the pandemic has also been linked to increased maternal depression and anxiety. This editorial emphasizes the need for increased surveillance in educational settings and early behavioral assessments by pediatricians. Further research is required to understand the long-term effects of maternal SSRIs. This knowledge can aid in timely interventions to protect the well-being of children born during COVID-19.

## Editorial

There are many maternal medications that cross the placenta and are known to have detrimental effects on the fetus. Some of them cause serious malformations, and these include vitamin A, ACE inhibitors, warfarin, and lithium [1]. Chloramphenicol is known to cause Gray Baby Syndrome, characterized by abdominal distension and hemodynamic collapse [1]. Other drugs, like opiates, are known to cause neonatal withdrawal syndrome [1]. Medications such as selective serotonin reuptake inhibitors (SSRIs) can also cause neonatal withdrawal syndrome [2]. In addition, there is a higher frequency of admission to a special care nursery for infants exposed to SSRIs during the third trimester [3]. It is unclear whether medications such as SSRIs have long-term effects on children with fetal exposure. These effects may include, but are not limited to: internalizing and externalizing behaviors, a change in IQ, cognitive changes, anxiety symptoms, and childhood depression.

Anxiolytics (benzodiazepines) are commonly used medications in depressed and anxious populations. Although not contraindicated in pregnancy, maternal use of anxiolytics has resulted in postnatal changes. Benzodiazepines have been shown to cause sedation, withdrawal symptoms, and floppy baby syndrome, all of which have detrimental effects on the developing infant [3]. Additionally, the withdrawal syndrome seen with maternal benzodiazepine use is significantly greater than that seen with maternal SSRI use [3]. It is important to consider that many mothers often take benzodiazepines and SSRIs as a combination therapy, thus increasing the risk of adverse effects on the developing fetus/child. During the height of the COVID-19 pandemic, social isolation played a large role in the increased incidence of depression and anxiety, and therefore, subsequent use of antidepressant and anxiolytic drugs increased linearly [4]. Moreover, previous research established that social isolation is known to have detrimental effects on mother and child [5].

We conducted a literature review. NCBI and PubMed were searched using the following words: ‘SSRI,’ ‘fetal development,’ ‘anxiolytics,’ ‘antidepressants,’ ‘congenital malformations,’ ‘contraindicated,’ ‘pregnancy,’ ‘fetus,’ ‘substance use,’ ‘social isolation,’ and ‘maternal depression.’ Elicit: The AI Research Assistant was prompted using the following phrases: ‘the effects of medication during pregnancy and its effects on the fetus,’ ‘antidepressant fetal withdrawal syndrome,’ ‘maternal medications and their effect on fetal development,’ ‘maternal use of SSRIs on fetal development.’ The references resulting from these searches were reviewed and analyzed for their relevance.

Medical hypothesis

Fetuses exposed to medications such as SSRIs and benzodiazepines, maternal mental illness, and social isolation due to the COVID-19 pandemic will demonstrate long-term multiplicative behavioral changes later in life. Evidence has shown that maternal use of SSRIs and benzodiazepines, especially during the second and third trimesters of pregnancy, can result in the fetus developing anxiety symptoms, childhood depression, and internalizing and externalizing behaviors [3,6]. Additionally, social isolation due to the COVID-19 pandemic increases the chances of depression, anxiety, sadness, and guilt.

SSRI effects on brain development

In the mouse, SSRI effects include an increase in the volume of the amygdala and insula and increased connectivity between these regions [7]. In humans, SSRI effects include a thick left lateral occipital cortex, a larger surface area of the left superior parietal cortex [8], changes in the microstructure of the right amygdala [9], and altered neural plasticity in the hippocampus due to an increase in BDNF expression [10].

Known effects of SSRIs on different regions of the brain

SSRIs are known to help improve an individual's mood, which is linked to the medication’s effects on the dorsal raphe nuclei [11]. Moreover, the effects of trans-placental SSRIs on fetal brain development are unclear. ​

Postnatal maternal benzodiazepine effects

Third-trimester benzodiazepines are known to have the following postnatal effects on the neonate: hypertonia, hyperreflexia, excessive crying, tremors, bradycardia, restlessness, irritability, seizures, abnormal sleep patterns, cyanosis, “Floppy Baby syndrome” (characterized by: hypothermia, muscular hypotonia, and low Apgar scores) [3]. These effects may take a longer amount of time to subside, depending on the amount and length of time of exposure in utero. It is also believed that anxiolytics (benzodiazepines) have a greater withdrawal symptom than that resulting from SSRIs [3].

Social isolation effect on neonates

Three years after the COVID-19 pandemic, we are beginning to see the effects of social isolation on populations. In particular, these effects are likely to be exacerbated in mothers and their infants born during the pandemic. These changes are manifested by the increased use of antidepressants in mothers [5]. Historically, there has been a significant social impact of institutionalization/social isolation on children in a post-Soviet country [12]. Similarly, the social isolation experienced by migrant mothers has been shown to negatively impact their children [13]. These negative effects will likely manifest in children born during the COVID-19 pandemic.

The effects of maternal medications on the fetus, in conjunction with mental illness and social isolation, have not yet been explored post-pandemic. Additional factors, such as increased rates of maternal mental illness and social isolation, have likely potentiated the neurobehavioral effects of these medications on children born during the pandemic.

To evaluate the aforementioned effects, we propose increased surveillance for behavioral changes in educational settings. This would include increased teacher/staff training to recognize these changes. The children identified as “at risk” for negative outcomes should be referred to behavioral counseling and other early-childhood interventions in order to prevent future problems as they grow older. We encourage pediatricians and other early childhood caretakers to complete behavioral assessments prior to school age. ​

Although the pandemic likely introduced many confounding variables, we believe additional studies are still potentially valuable. Of course, such studies may have limitations due to the many additional contextual factors related to the pandemic.

We recommend additional research pertaining to the long-term behavioral effects of maternal SSRIs on these cohorts. Controls may be difficult to select due to the global impact of COVID-19; however, controls could be identified using siblings born before the pandemic. It is critical to understand these implications in order to prevent further detrimental effects on the children born during the pandemic.

This editorial is written with the intention of disseminating these ideas to help individuals caring for children recognize potentially affected children. Children may then benefit from interventions intended to mitigate the adverse effects associated with the COVID-19 pandemic.
